# Supplementary material for: Predicting visual function by interpreting a neuronal wiring diagram
Source: Nature. 2024 Oct 2;634(8032):113–23. doi: 10.1038/s41586-024-07953-5 (PMC11446822; doi:10.1038/s41586-024-07953-5)
Supplement: Supplementary file 6 — CRF and ERF predictions for individual TmY4 and TmY9 cells. Analogous to Supplementary Data 3, but for TmY target types. Shown are the top four monosynaptic pathways, the strongest pathway passing through each of the top ten intermediary types (ranking from Extended Data Fig. 7), and the trisynaptic pathway Tm1–TmY–Dm3–TmY (see the section entitled Prediction of spatial normalization). [file 41586_2024_7953_MOESM6_ESM.zip › DataS4/TmY9q/720575940632113706.pdf]

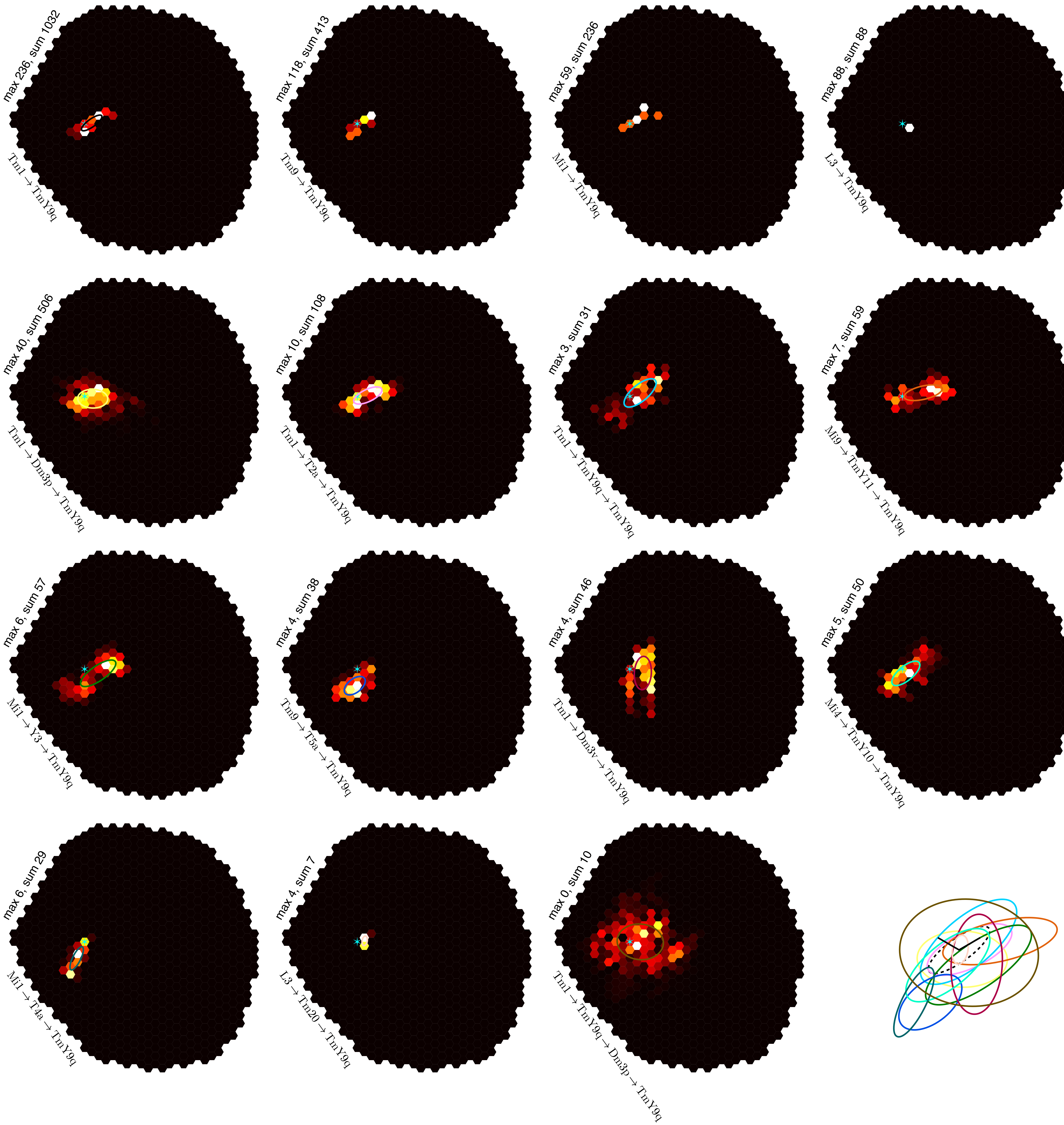
max 236, sum 1032
Tm1 → TmY9q
max 118, sum 413
Tm9 → TmY9q
max 59, sum 236
Mi1 → TmY9q
max 88, sum 88
L3 → TmY9q
max 40, sum 506
Tm1 → Dm3p → TmY9q
max 10, sum 108
Tm1 → T2a → TmY9q
max 3, sum 31
Tm1 → TmY9q → TmY9q
max 7, sum 59
Mi9 → TmY11 → TmY9q
max 6, sum 57
Mi1 → Y3 → TmY9q
max 4, sum 38
Tm9 → T5a → TmY9q
max 4, sum 46
Tm1 → Dm3v → TmY9q
max 5, sum 50
Mi4 → TmY10 → TmY9q
max 6, sum 29
Mi1 → T4a → TmY9q
max 4, sum 7
L3 → Tm20 → TmY9q
max 0, sum 10
Tm1 → TmY9q → Dm3p → TmY9q
